# Molecular epidemiology of paediatric respiratory adenovirus infections in Île-de-France: findings from whole-genome sequencing

**DOI:** 10.1093/ve/veag064

**Published:** 2026-09-26

**Authors:** Maud Salmona, Elise Diaz, Linda Feghoul, Abdeljalil Senhaji Rachik, Héloïse Delagrèverie, Honorine Fenaux, Aurélie Schnuriger, Audrey Baron, Lamia Limam, Séverine Mercier-Delarue, Elyanne Gault, Jérôme LeGoff

**Affiliations:** Virology Department, Hôpital Saint-Louis, AP-HP, Université Paris Cité, 1 av Claude Vellefaux, 75010 Paris, France; Université Paris Cité, Université Paris Cité, Biology and Pathogenesis of Viral Infection Team, INSERM UMR 1342, Saint-Louis Research Institute, 1 av Claude Vellefaux, 75010 Paris, France; Université Paris Cité, Université Paris Cité, Biology and Pathogenesis of Viral Infection Team, INSERM UMR 1342, Saint-Louis Research Institute, 1 av Claude Vellefaux, 75010 Paris, France; Virology Department, Hôpital Saint-Louis, AP-HP, Université Paris Cité, 1 av Claude Vellefaux, 75010 Paris, France; Virology Department, Hôpital Saint-Louis, AP-HP, Université Paris Cité, 1 av Claude Vellefaux, 75010 Paris, France; Virology Unit, Department of Microbiology, Hôpital Avicenne, AP-HP, Bobigny France; Université Sorbonne Paris Nord and Université Paris Cité, INSERM UMR 1137, IAME, 125 rue de Stalingrad, 93000 Bobigny, France; Virology Department, Hôpital Paul-Brousse, AP-HP, 2 avenue Paul-Vaillant-Couturier, 94800 Villejuif, France; Department of Virology, Hôpitaux Saint-Antoine, Tenon, Trousseau, AP-HP, Sorbonne Université, 184 rue du Faubourg Saint-Antoine, 75012 Paris, France; Microbiology Laboratory, Hôpital Robert-Debré, AP-HP, Université Paris Cité, 48 boulevard Sérurier, 75019 Paris, France; Virology Department, Hôpital Paul-Brousse, AP-HP, 2 avenue Paul-Vaillant-Couturier, 94800 Villejuif, France; Virology Department, Hôpital Saint-Louis, AP-HP, Université Paris Cité, 1 av Claude Vellefaux, 75010 Paris, France; Microbiology Laboratory, Hôpital Ambroise-Paré, AP-HP, 9 avenue Charles de Gaulle, 92100 Boulogne-Billancourt, France; Virology Department, Hôpital Saint-Louis, AP-HP, Université Paris Cité, 1 av Claude Vellefaux, 75010 Paris, France; Université Paris Cité, Université Paris Cité, Biology and Pathogenesis of Viral Infection Team, INSERM UMR 1342, Saint-Louis Research Institute, 1 av Claude Vellefaux, 75010 Paris, France

**Keywords:** human adenovirus, paediatrics, respiratory infections, whole genome sequencing, genomic surveillance

## Abstract

Background: Human adenoviruses (HAdVs) are a frequent cause of respiratory tract infections in children; however, their epidemiological patterns and genomic diversity remain insufficiently characterized in Europe. As respiratory HAdVs can cause outbreaks of acute and severe disease and show periodic re-emergence, improved understanding of their circulation is needed. Aim: To investigate the epidemiology, age distribution, and genomic diversity of respiratory HAdVs in paediatric patients in Île-de-France during the first half of 2022. Methods: We retrospectively analysed 6 956 respiratory samples from patients < 18 years, collected at five hospitals (Jan–June 2022). HAdVs were detected by multiplex PCR. Viral loads were quantified in mono-infections, and whole-genome sequencing (WGS) was performed on samples with sufficient viral load. Results: HAdVs were detected in 14% of samples, making them the second most prevalent virus after rhinovirus/enterovirus. Co-infections were frequent (64%), particularly in younger children. Among the 254 mono-infected samples with viral-load quantification, 110 (43%) had low or undetectable viral loads. WGS of 122 samples revealed HAdV species B (62.3%), C (34.4%), and F (3.3%). Species distribution varied by age: HAdV-C predominated in children under 2 years, while HAdV-B was more common in older children. The most frequent genotypes were B3 (76.3%), B66 (18.4%), and B7d (5.3%) within species B, and C1 (33.3%), C89 (26.2%), and C2 (23.8%) within species C. Conclusion: HAdVs are prevalent among children with respiratory infections, with age-specific patterns and substantial genomic diversity. The circulation of diverse genotypes, including recombinants and potentially more virulent variants, highlights the importance of sustained molecular surveillance.

## Introduction

Human adenoviruses (HAdVs) are non-enveloped viruses with double-stranded DNA genomes of approximately 35 kilobase pairs. They belong to the family *Adenoviridae*, genus *Mastadenovirus,* and are classified into seven species (A–G), comprising more than100 distinct types, as designated by the HAdV Working Group (http://hadvwg.gmu.edu/). HAdVs exhibit diverse tissue tropisms and are responsible for a broad spectrum of infections, including keratitis, respiratory illnesses, encephalitis, cystitis, and gastrointestinal disorders. Most of these infections are benign and self-limiting. Species A, B, C, and E are mostly associated with respiratory infections, while species B, D, and E are the main cause of conjunctival disease. Species F is the major cause of adenovirus-related gastroenteritis, whereas species G has only rarely been associated with gastrointestinal disease (Lion 2014).

In the respiratory setting, HAdVs are linked to a spectrum of clinical manifestations, ranging from mild to severe, sometimes fatal conditions. Outbreaks have been documented in diverse settings, including military camps, prisons, hospitals, and community gatherings, affecting both adults and children. Specific genotypes, such as HAdV-B3, -B7, -B14, -B55, and -E4, have frequently been associated with acute, severe respiratory disease outbreaks (Kajon et al. 2019). These viruses show periodic re-emergences, often resulting in localized epidemics and the subsequent use of targeted vaccines, such as the oral E4 and B7 vaccines administered to U.S. military personnel (Kajon, Lamson, and St. George 2019). Additionally, HAdV-B55 has been implicated in serious epidemics across Asia and the United States, with sporadic cases also reported in France and other European countries (Lafolie et al. 2016, Hang et al. 2020, Mohamedi et al. 2025).

Other incidents further underscore the critical need for robust epidemiological surveillance of HAdVs. In April 2022, the United Kingdom experienced an outbreak of paediatric hepatitis of unknown aetiology, in which 65% of cases tested positive for HAdV (Morfopoulou et al. 2023). The most frequently identified type was HAdV-F41, typically associated with acute community-acquired gastroenteritis, but not previously recognized as a cause of hepatitis. Co-infection with adeno-associated virus type 2 (AAV2) was frequently observed. It has been hypothesized that viral co-infection in seronegative individuals with specific genetic predispositions (*e.g.* HLA-DRB1^*^04:01) may trigger an abnormal immune response leading to hepatic cytolysis (Morfopoulou et al. 2023, Liddy et al. 2025). The concurrent circulation of HAdV-F41 and AAV2 may therefore represent the underlying cause of this outbreak. These epidemiological events highlight the evolving nature of HAdV infections and underscore the need for ongoing epidemiological surveillance of HAdV-related diseases.

Respiratory HAdVs are particularly prevalent in young children, accounting for approximately 5%–10% of all paediatric respiratory infections (Mengelle et al. 2014, Jain et al. 2015, Tabatabai et al. 2023). Despite their clinical impact, HAdVs have not been studied as extensively as other respiratory viruses, such as influenza and respiratory syncytial virus (Fourati and Loubet 2025). Furthermore, a notable increase in the circulation of HAdV was reported in the United States at both national and local levels (Abdullah et al. 2023, Respiratory Adenovirus National Trends—NREVSS | CDC 2024).

The aim of this study was to gather and analyse epidemiological data on the respiratory HAdVs circulating among paediatric populations in the Île-de-France region, employing whole-genome sequencing (WGS) for detailed strain characterization and monitoring of viral trends.

## Materials and methods

### Study design and sampling

We conducted a retrospective study at five healthcare centers across Île-de-France, including 6 956 upper respiratory tract samples (nasopharyngeal swabs or oropharyngeal aspirations) collected from paediatric patients between January and June 2022. All samples were obtained as part of routine clinical care from patients younger than 18 years of age. Samples were not randomly selected and were not enriched for specific symptoms, clinical presentations, or treatments. The flow chart illustrating the sample selection process for this study is shown in Supplementary Fig. S1.

This study involved a retrospective analysis of anonymized data collected in the context of routine clinical care. As such, it does not fall under the scope of the French Jardé law, which regulates research involving human subjects. All data were processed in compliance with applicable data protection and privacy regulations. No additional interventions or procedures were performed for the purpose of this study, and patient confidentiality was strictly maintained throughout the research process. In accordance with French legislation, ethics committee approval was not required for this type of study.

### Viral detection and quantification

Respiratory samples were analysed using multiplex PCR panels according to routine diagnostic procedures and manufacturers’ instructions. One healthcare center used the Allplex™ Respiratory Panel (Seegene Inc.), whereas the four remaining centers used the BioFire® FilmArray RP2 panel (bioMérieux). For samples identified as HAdV mono-infections, viral DNA quantification was subsequently performed using the HAdV R-GENE® PCR kit (bioMérieux), following the manufacturer’s recommendations. The quantification range was 500 copies/mL to 9 log_10_ copies/mL. Values above the upper limit of quantification were capped at 9 log_10_ copies/mL for the primary analysis, as residual sample material was unavailable for dilution and re-quantification. Analyses using uncapped instrument-reported values above this threshold were considered exploratory sensitivity analyses, and these values should not be interpreted as fully quantitative measurements.

### Whole genome sequencing

Samples with HAdV DNA concentrations exceeding 500 copies/mL underwent whole-genome sequencing. Total nucleic acids were extracted from 1 mL of sample using the NucliSENS easyMAG system (bioMérieux, Marcy l’Étoile, France) and eluted in 60 μL. Extracted nucleic acids were subsequently concentrated to a final volume of 20 μL using the Zymo DNA Clean & Concentrator Kit (Zymo Research). Sequencing libraries were prepared using the Agilent SureSelect XT HS Library Preparation Kit. HAdV-specific target enrichment was performed using the SureSelect XT Community Design Pan Adenovirus probes (5191–6 713), according to the manufacturer’s instructions (Houldcroft et al. 2018). After normalization, libraries were pooled and sequenced on an Illumina NextSeq 500 platform using a v2 2 × 150 bp cartridge.

### Bioinformatics analysis

Post-sequencing, raw data were processed using a custom bioinformatics pipeline. This pipeline included read trimming and filtering using Trimmomatic, followed by mapping of reads with Bowtie2 to a set of reference sequences from GenBank. The reference sequence providing the best genome coverage was selected for downstream analysis. Consensus sequences were then generated for each sample using iVar.

Phylogenetic analysis was conducted using the MAFFT alignment tool and IQ-TREE2, employing the Maximum Likelihood method with model K2 + G + I, supported by 1 000 bootstrap replicates. Phylogenetic trees were visualized using iTOL.

All consensus sequences generated in this study were deposited on Genbank (accession numbers: PX575684—PX575805).

Intrahost single nucleotide variants (iSNVs) were analysed from iVar variant-calling outputs generated from reference-based whole-genome sequencing. Minor allele frequency (MAF) was defined as the number of reads supporting the minor (non-consensus) allele at a given position divided by the total read depth at that position. Samples were included in the iSNV analysis if they had a mean sequencing depth ≥ 100× against the genotype-specific reference. Variants were retained if they passed iVar’s default quality filter (PASS = TRUE), had a MAF ≥5% and < 50%, and a site depth ≥ 100×; insertions and deletions were excluded. No unique molecular identifiers (UMIs) or molecular barcodes were incorporated during library preparation; consequently, PCR/enrichment duplicates could not be identified using barcode-based deduplication, and duplicate reads were not removed prior to variant calling. iSNV incidence was calculated as the number of iSNVs per kilobase of genome sequence. Nucleotide diversity (π) was estimated for each sample as π = Σ[2p(1 − p)]/L, where p is the minor allele frequency at a given site and L is the genome length analysed. Genome-wide iSNV distribution was assessed for each genotype using a chi-squared goodness-of-fit test on iSNV counts in 500-bp bins, assuming a uniform expected distribution. No adjustment was made for sequencing depth or callable genome length, and ITRs were retained.

### Statistical analysis

All analyses were performed using R software (RStudio, version 2023.03.1 + 446). Data preprocessing and recoding were conducted using the tidyverse package suite, including dplyr and lubridate. Summary tables were generated using gtsummary and gt, and figures were produced with ggplot2 and ggpubr. Categorical variables were reported as counts and percentages and compared using Pearson’s chi-squared test or Fisher’s exact test, as appropriate. Continuous variables were summarized as medians with interquartile ranges (IQR) and compared using the Wilcoxon rank-sum test. Viral load and age distributions were visualized using boxplots with jittered observations. Pairwise comparisons of viral load and age across groups were performed using Wilcoxon rank-sum tests with Bonferroni correction for multiple testing, whereas pairwise comparisons of intrahost diversity metrics (nucleotide diversity and iSNV counts) across HAdV species were performed using Wilcoxon rank-sum tests with Benjamini–Hochberg correction. Statistical significance was defined as a two-sided *P*-value < .05.

## Results

### Epidemiological analysis using multiplex panel

The main findings from the multiplex respiratory panel testing are presented in Table 1. Overall, 57.6% (n = 4006/6956 samples) of the analysed samples contained at least one respiratory virus. HAdV was detected in 971 samples (14.0%), making it the second most frequently identified virus following rhinovirus/enterovirus, which was found in 29% (n = 2006) of samples. HAdV detection rates differed significantly between the two multiplex assays used during the study period, with 11.1% (n = 150/1351) positive samples using the Allplex™ Respiratory Panel and 14.6% (n = 821/5605) using the BioFire FilmArray RP2 panel (*P* < .001). Other viruses detected included endemic coronaviruses (4.3%, n = 302), Bocavirus (8.6%, n = 116 among the 1 351 samples tested with the Allplex™ respiratory panel), Parainfluenza viruses (3.7%, n = 258), Influenza viruses A/B (11%, n = 769), Respiratory Syncytial Virus (2.8%, n = 193), Metapneumovirus (3.3%, n = 229), and SARS-CoV-2 (7.5%, n = 525). Additionally, 16% (n = 1 107) of the samples contained more than one virus. No significant seasonal variation in HAdV detection was observed during the study period (Fig. 1).

**Table 1:** Comparison of clinical and virological characteristics between HAdV DNA-positive and -negative samples.

	**Overall (N = 6 956)** ^***1***^	**HAdV DNA—(N = 5 985)** ^***1***^	**HAdV DNA + (N = 971)** ^***1***^	***P*-value** ^***2***^
**Other detected Viruses**				
Endemic Coronavirus	302 (4.3%)	250 (4.2%)	52 (5.4%)	0.095
Bocavirus^#^	116 (8.5%)	85 (7.1%)	31 (21%)	<0.001
Parainfluenza Virus	258 (3.7%)	186 (3.1%)	72 (7.4%)	<0.001
Influenza Virus	769 (11%)	668 (11%)	101 (10%)	0.5
RSV	193 (2.8%)	166 (2.8%)	27 (2.8%)	>0.9
Human Metapneumovirus	229 (3.3%)	184 (3.1%)	45 (4.6%)	0.011
Rhinovirus/Enterovirus	2006 (29%)	1 607 (27%)	399 (41%)	<0.001
SARS-CoV-2	525 (7.5%)	445 (7.4%)	80 (8.2%)	0.4
**Viral Co-infection**	**1 107 (16%)**	**486 (8.1%)**	**621 (64%)**	**<0.001**
Number of Viruses Detected				
2 viruses	901 (13%)	425 (7.1%)	476 (49%)	
3 viruses	167 (2.4%)	53 (0.9%)	114 (12%)	
≥ 4 viruses	40 (0.6%)	9 (0.1%)	31 (3.2%)	
**Age (years) median (Q1, Q3)**	**2.0 (0.0, 6.0)**	**2.0 (0.0, 7.0)**	**1.0 (0.0, 3.0)**	**<0.001**
**Month of Sampling**				
January	1 617 (23%)	1 436 (24%)	181 (19%)	
February	1 219 (18%)	1 077 (18%)	142 (15%)	
March	1 471 (21%)	1 298 (22%)	173 (18%)	
April	1 450 (21%)	1 200 (20%)	250 (26%)	
May	1 191 (17%)	971 (16%)	220 (23%)	
June	8 (0.1%)	3 (<0.1%)	5 (0.5%)	

^*^1^*^n (%)

^*^2^*^Pearson’s Chi-squared test; Wilcoxon rank sum test

# Bocavirus detection was available only for samples tested with the Allplex™ respiratory panel (n = 1 351), including 150 HAdV DNA-positive and 1 201 HAdV DNA-negative samples.

Data are presented as *n (%)* for categorical variables and *median (Q1–Q3)* for continuous variables. Statistical comparisons were performed using Pearson’s Chi-squared test for categorical variables and the Wilcoxon rank sum test for continuous variables.

**Figure 1:**
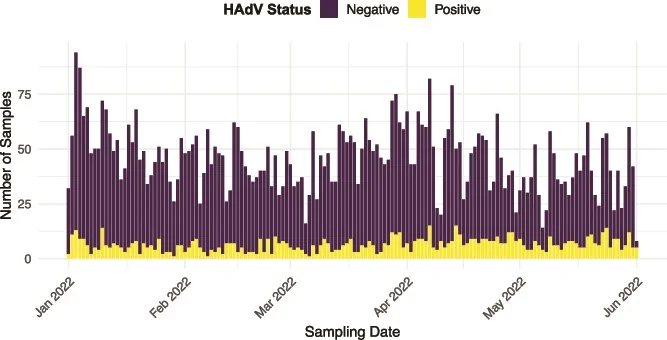
Frequency of human adenovirus (HAdV) positive and negative samples by sampling date. Stacked bars represent the number of samples analysed each day, colour-coded by HAdV status. No significant seasonal variation in HAdV occurrence was observed during the study period (January–June 2022).

### Characteristics of HAdV positive samples

Children with HAdV infection were younger (median age 1 year, Interquartile Range [IQR]: 0–3 years) compared to those without HAdV infection (median age 2 years, IQR: 0–7 years, *P* < .001). Among HAdV-positive samples, 64% (n = 621/971) also contained another virus. The detection frequencies of parainfluenza viruses, metapneumovirus, and rhino/enterovirus was significantly higher in these co-infected samples (*P* < .001) (Table 1). Additionally, children with co-infections were younger than those with HAdV mono-infection (median age 1 year, IQR: 0–2 years *versus* median age 2 years, IQR: 1–4 years, *P* < .001).

### Viral load in HAdV mono-infection

HAdV DNA quantification was performed for 254 samples of the 350 samples with HAdV mono-infection, due to limited sample availability for the remaining specimens. HAdV DNA was undetectable in 53 samples and detectable but not quantifiable (below 500 copies/mL) in 57 samples. Age did not differ significantly between the 206 non-quantifiable samples (HAdV DNA undetectable, detectable but < 500 copies/mL, or unavailable for quantification [n = 96]) and the 144 quantifiable samples (median 2.0 years [IQR, 0.4–6.0] versus 2.3 years [IQR, 1.1–4.2]; *P* = .13). Among the 144 samples with quantifiable HAdV loads (over 500 copies/mL), the median load was 6.8 log_10_ copies/mL (IQR: 4.5–8.4). Of these, 24 (16.7%) reached the upper limit of quantification (9 log10 copies/mL) and were capped at this value for the primary analysis. Viral loads did not differ significantly across age groups (<2, 2–5, and > 5 years) (Supplementary Fig. S2), and results were unchanged in a sensitivity analysis using the uncapped values (Supplementary Fig. S3).

### Whole genome sequencing and epidemiological typing

Capture enrichment NGS for whole genome sequencing was performed on the 144 quantifiable samples, achieving a median of 473 000 reads per sample and satisfactory genome coverage (>90% of the genome covered at ≥10×) for 122 samples. Comparison of epidemiological and virological characteristics across the main sample selection steps is summarized in Supplementary Table S1. The 122 samples with successful WGS and the 22 with WGS failure did not differ significantly in age (median 2.3 years [IQR, 1.1–4.0] versus 2.0 years [IQR, 1.0–6.0]; *P* = .18) or healthcare center/multiplex assay distribution (*P* = .18). In contrast, viral load was significantly lower in samples with WGS failure (median 3.43 log10 copies/mL [IQR, 3.12–4.05] versus 7.20 log10 copies/mL [IQR, 6.05–8.51]; *P* < .001).

Sequencing quality metrics for the 122 samples are provided in Supplementary Table S2. Median genome coverage was 99.8% (IQR: 99.7–100.0), median mean sequencing depth was 1 409× (IQR: 463–3 439), and median duplicate read percentage was 70.7% (IQR: 55.2–79.1). Median genome-wide depth exceeded 1 200× for HAdV-B and HAdV-C (≥99% of positions above the 100× iSNV threshold), *versus* 66× for HAdV-F (14% of positions ≥100×; Supplementary Fig. S4). Typing results *via* phylogenetic analysis of whole genome sequencing identified several species: 76 samples of HAdV-B (62.3%), 42 of HAdV-C (34.4%), and 4 of HAdV-F (3.3%) (Fig. 2A and 2B). The distribution of genotypes identified in the samples by species is represented in Fig. 2B. Among the HAdV-B species, the predominant type was HAdV-B3, representing 76.3% (58/76) of the sequenced HAdV-B samples. Other types identified included HAdV-B7 (5.3%, 4/76) and HAdV-B66, a HAdV recombinant HAdV-B3/B7 strain (18.4%, 14/76). Notably, among the HAdV-B7, all strains clustered within the same clade as HAdV-B7d (bootstrap =100%), a variant associated with severe respiratory outbreaks in the USA and Asia (Zhao et al. 2014, Scott et al. 2016). Regarding HAdV-C, the most frequently identified type was HAdV-C1 (33.3%, 14/42). Other genotypes identified were HAdV-C2 (23.8%, 10/42), C5 (11.9%, 5/42), C89 (26.2%, 11/42) and C104 (4.8%, 2/42). For HAdV-F species, only genotype HAdV-F41 was detected (100%, 4/4). The median HAdV load for HAdV-B positive samples (7.86 log_10_ copies/mL, IQR: 7.02–9.00) was significantly higher than for HAdV-C (6.14 log_10_ copies/mL, IQR: 4.34–7.01, adjusted *P* < .001). The median viral load for HAdV-F positive samples (3.59 log_10_ copies/mL; IQR 3.38–4.50) was significantly lower than for HAdV-B (adjusted *P* < .001) and HAdV-C (adjusted *P* = .012) (Fig. 3). Among the 24 samples exceeding the upper limit of quantification, 20 were HAdV-B, one was HAdV-C, and three could not be assigned to a species because WGS was unsuccessful. Using uncapped instrument values for these samples (range, 9.01–10.96 log_10_ copies/mL) yielded similar results, with no change in species-specific median viral loads or in the significance of the comparisons between species (Supplementary Fig. S3). The median ages of children infected with different HAdV species varied. Children infected with HAdV-B were older than those infected with HAdV-C (3.26 years, IQR 1.62–4.45 versus 1.46 years, IQR 0.97–2.46; adjusted *P* = .0011). There was no significant difference between HAdV-B and HAdV-F (3.26 years, IQR 1.62–4.45 versus 0.82 years, IQR 0.40–3.11; adjusted *P* = .14), or between HAdV-C and HAdV-F (1.46 years, IQR 0.97–2.46 versus 0.82 years, IQR 0.40–3.11; adjusted *P* = .27) (Fig. 4).

**Figure 2:**
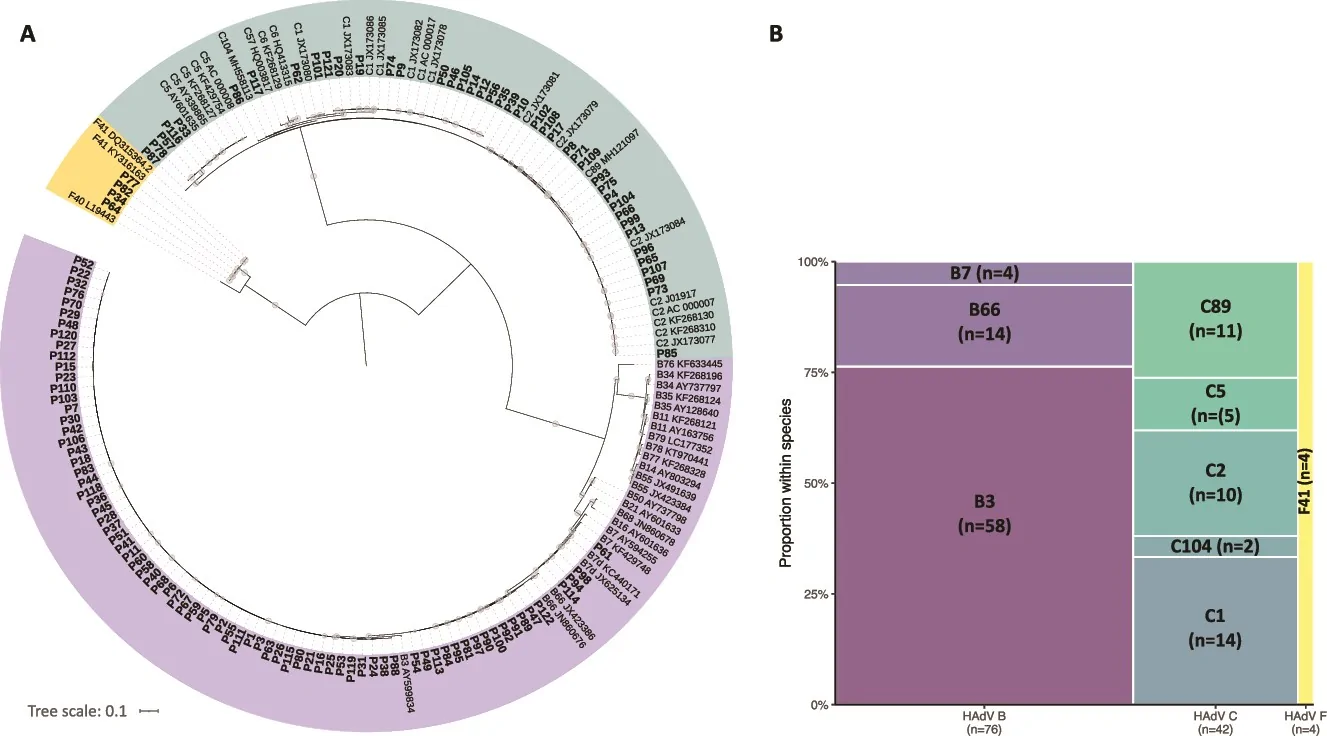
Phylogenetic analysis and genotype distribution of respiratory human adenoviruses (HAdV) detected in this study. (A) Phylogenetic tree of HAdV whole-genome consensus sequences obtained in this study. Bootstrap values above 80% are indicated with grey circles. HAdV consensus sequences generated from analysed samples are highlighted in bold. Reference sequences from GenBank are included for comparison. (B) Mosaic plot representing genotype distribution within each HAdV species. The width of each block is proportional to the number of samples belonging to each species, and the height of each segment represents the relative proportion of each genotype within the corresponding species.

**Figure 3:**
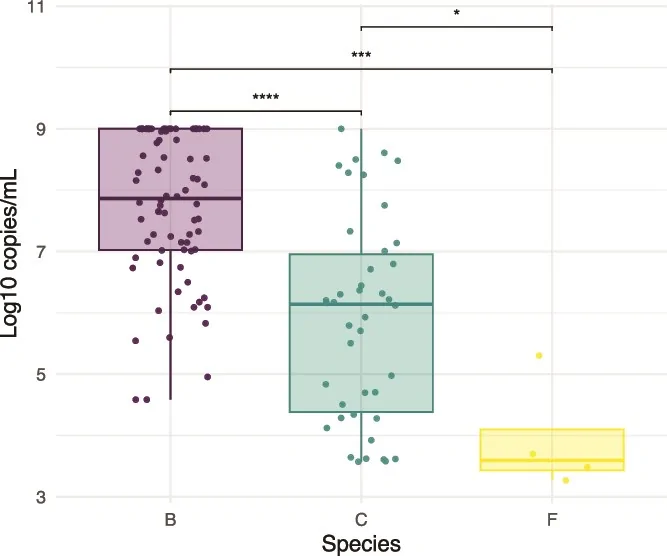
Distribution of human adenovirus (HAdV) DNA viral load (log_10_ copies/mL) by HAdV species. Boxplots show the distribution of quantifiable HAdV loads (Log_10_ copies/mL) in samples stratified by HAdV species. Individual data points are overlaid with jitter. The horizontal line within each box indicates the median viral load. Values at or above 9 log_10_ copies/mL correspond to the upper limit of quantification of the assay, viral loads above this threshold were capped at 9 log_10_ copies/mL. Statistical comparisons between groups are marked by asterisks, where *P* < .05 is denoted by ^*^, *P* < .01 by ^**^, *P* < .001 by ^***^, and *P* < .0001 by^****^.

**Figure 4:**
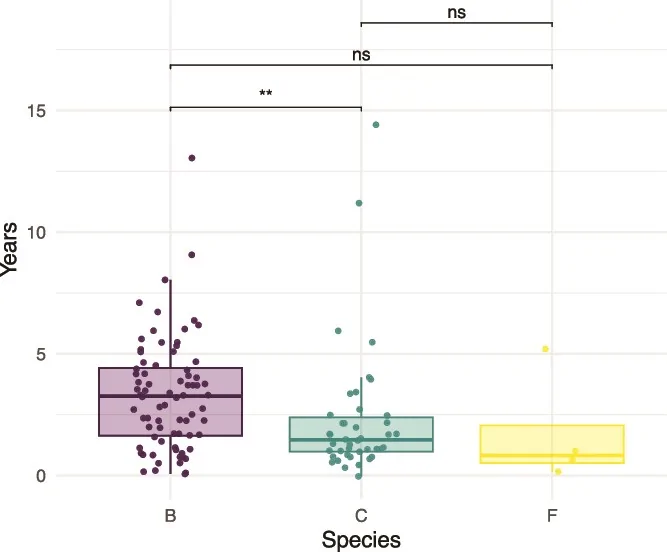
Distribution of age (years old) by human adenovirus (HAdV) species. Boxplots show the distribution of patient ages old among samples stratified by HAdV species. Individual data points are overlaid with jitter to display variability. The horizontal line within each box represents the median age. Statistical comparisons between groups are indicated by asterisks, where *P* < .05 is denoted by ^*^, *P* < .01 by ^**^, *P* < .001 by ^***^, *P* < .0001 by ^****^ and non-significant differences (*P* ≥ 0.05) are indicated as ‘ns’.

### Intrahost diversity analyses

Among the 122 samples with successful WGS, 5 (1 HAdV-B7d, 1 HAdV-C1, 2 HAdV-C2, and 1 HAdV-C89) had a mean sequencing depth below the ≥100× threshold required for iSNV analysis (range, 22.9–42.6×) and were therefore excluded, leaving 117 samples for intrahost diversity analyses. Among the 19 573 retained iSNVs, median site depth was 165× (IQR, 127–238) and median minor-allele support was 12 reads (IQR, 9–21). Overall, 28.9% of iSNVs were supported by fewer than 10 minor-allele reads, and 71.3% had minor-allele support from only one strand (Supplementary Table S3). The median number of iSNVs was 50 per sample overall. HAdV-C samples showed a significantly higher number of iSNVs compared with HAdV-B samples (median 72 [IQR 35.8–116.0] versus 43.5 [IQR 33.0–61.2], adjusted *P* = .032), whereas no significant differences were observed between HAdV-F and the other species. Similarly, nucleotide diversity (π) tended to be higher in HAdV-C samples (median 3.30 × 10^−4^ [IQR 1.99 × 10^−4^–9.74 × 10^−4^]) compared with HAdV-B (median 2.69 × 10^−4^ [IQR 1.94 × 10^−4^–4.14 × 10^−4^]) and HAdV-F (median 2.73 × 10^−5^ [IQR 2.01 × 10^−5^–1.48 × 10^−4^]), although these differences did not reach statistical significance after multiple testing correction. Genome-wide mapping showed that iSNVs were not uniformly distributed but clustered at discrete positions (goodness-of-fit test, *P* < .001 for each genotype; Supplementary Fig. S5). Recurrence profiles differed between species. In HAdV-B (n = 75), a few regions recurred in a majority of samples: E3 (up to 62/75), the L2 penton/core unit (54/75), the non-coding VA RNA locus (43/75) and L4 100K (41/75). In HAdV-C (n = 38), clustering was weaker (maximum recurrence 9/38) and involved mainly the fibre, E4, penton base and E3 regions, with no recurrent position at the VA RNA locus (Supplementary Table S4). Variation within hexon was dispersed rather than peaked in both species. Recurrent positions had higher median sequencing depth than sporadic positions (218× versus 156×; *P* < .001). Variant-level sequencing and strand-support metrics for all retained iSNVs are provided in Supplementary Table S5. Inverted terminal repeats, accounted for 7% of recurrent positions in HAdV-B and 41% in HAdV-C To assess possible mixed viral populations, MAF distributions were examined at the individual-sample level. MAFs were predominantly low (median, 6.8%; IQR, 5.7–9.1%), with 79.5% of iSNVs between 5% and 10%. Most samples showed a low-frequency-dominated distribution, whereas seven of 117 (6.0%), predominantly HAdV-C samples, showed a broader distribution at intermediate frequencies (15–40%).

## Discussion

Our study, conducted in a large paediatric cohort in the Paris area, confirms that HAdVs are an important cause of respiratory infections in children (Jain et al. 2015), ranking as the second most frequently detected virus after rhinovirus/enterovirus during the study period. WGS of mono-infected samples identified species HAdV-B (62.3%) and HAdV-C (34.4%) as the predominant groups, each showing substantial genotype diversity.

The selection of HAdV-positive samples relied on two different multiplex respiratory panels used during the study period, namely the BioFire FilmArray RP2 and the Allplex™ Respiratory Panel assays. We observed a significant difference in HAdV positivity rates between the two assays in this study. The use of two different diagnostic assays may have introduced selection bias in HAdV detection. Previous evaluations reported reduced sensitivity of earlier FilmArray respiratory panel versions for HAdV detection, particularly in low viral load samples and for some genotypes, although specificity remained very high (Doern et al. 2013, Huang et al. 2018). The FilmArray RP2 version used in this study was subsequently redesigned to improve HAdV detection across species A–F. Comparative studies between FilmArray and Allplex respiratory panels have generally shown overall concordant HAdV detection (Chan et al. 2018, Lade et al. 2022). Differences in tested populations or local viral circulation may also have contributed to the observed differences in HAdV positivity rates.

Our data revealed distinct age-related trends in viral characteristics. HAdV-C infections and co-infections were significantly more frequent in younger children (<2 years), while HAdV-B infections were more common in older children. These patterns suggest age-related differences in susceptibility, immune response, or exposure risk, and are consistent with previous epidemiological studies (Abdullah et al. 2023, Probst et al. 2025).

The study period was selected to investigate HAdV circulation during 2022, a post-pandemic period marked by unusual epidemiological patterns and increased global surveillance of HAdV infections following reports of unexplained paediatric hepatitis associated with HAdV-F41 (Morfopoulou et al. 2023, Liddy et al. 2025). Disruptions in viral circulation and reduced population immunity have been associated with the re-emergence and increased severity of several respiratory pathogens (Nygaard et al. 2024, Munro and House 2026). Global surveillance data have demonstrated shifts in HAdV circulation patterns since the pandemic, including changes in dominant types and seasonal dynamics (Munro and House 2026, Papini et al. 2026). Such epidemiological changes may have contributed to the patterns observed in our cohort.

Although HAdVs were frequently detected, their clinical impact remains difficult to evaluate. In our cohort, 64% of HAdV-positive samples were co-infected with at least one other respiratory virus. Among the 254 mono-infected samples for which viral-load quantification was attempted, 110 (43%) had low or undetectable viral loads. This makes it challenging to determine the true contribution of HAdV to respiratory illness and highlights the need for integrated virological and clinical data to better assess its pathogenic role. Recent studies have explored the impact of HAdV co-infections with other respiratory pathogens in children (Niu et al. 2025, Piao et al. 2025). A systematic review and meta-analysis found that patients with viral, bacterial, or fungal co-infections experienced more severe clinical outcomes compared with those infected with HAdV alone (Niu et al. 2025). In children with *Mycoplasma pneumoniae* lobar pneumonia, co-infection with HAdV, especially when associated with high viral loads, was linked to increased disease severity compared to *M. pneumoniae* infection alone (Piao et al. 2025).

Notably, HAdV-B was associated with significantly higher viral loads than HAdV-C and HAdV-F. Although this may reflect higher viral replication in the respiratory tract, the cross-sectional nature of the analysis precludes firm conclusions, and other factors such as tissue tropism or timing of sampling may also contribute to the observed differences. This observation aligns with findings from the U.S., where high viral loads were reported in patients infected with HadV-B3, a genotype associated with severe disease (Abdullah et al. 2023). A previous study also reported that high adenoviral loads in nasopharyngeal aspirate (>6 log_10_ copies/mL) were strongly associated with symptomatic respiratory tract infection in hospitalized children, whereas lower viral loads may reflect prolonged shedding or incidental detection (Schjelderup Nilsen et al. 2019). While our study lacked clinical severity data, prior evidence has linked high adenoviral load to more severe clinical outcomes in hospitalized children under 9 years (Goikhman et al. 2020). These findings support the notion that viral load could be a helpful prognostic marker in paediatric respiratory illness and may aid differential diagnosis.

Previous studies have reported species-specific differences in clinical presentation and inflammatory responses in paediatric HAdV infections. In particular, HAdV-C infections have been associated with higher hospitalization rates and stronger inflammatory responses (Nakamura et al. 2018, Probst et al. 2025). Unfortunately, the absence of detailed clinical data in our cohort did not allow assessment of genotype-specific clinical outcomes.

The dominance of HAdV-B and particularly HAdV-B3 in our cohort is consistent with other studies (Kajon et al. 2019, Abdullah et al. 2023). HAdV-B66, a known recombinant of B3 and B7 (primarily named as HAdV-7 h), was the second most prevalent B type and has been previously associated with lower respiratory tract infections (Dehghan et al. 2012). Importantly, we also detected the HAdV-B7d variant, a highly virulent strain that has contributed to severe respiratory outbreaks with increased mortality in Asia and the U.S. (Zhao et al. 2014, Scott et al. 2016). Our identification of B66 and B7d reinforces the need for ongoing genomic monitoring to anticipate and detect strains with epidemic potential.

Among HAdV-C species, we observed five different genotypes. C1 and C89 were most common. HAdV-C89, recently described, is characterized by a unique penton base and combined hexon/fibre structure derived from HAdV-C2 (Dhingra et al. 2019), though its pathogenic potential remains poorly defined. We also detected C104, a recently described recombinant of C1 and C2 (Ji et al. 2021), previously found in a pneumonia case, highlighting the ongoing evolution within HAdV-C species and the need for further investigation.

Interestingly, we identified HAdV-F41, typically associated with gastrointestinal disease, in respiratory samples. These cases showed significantly lower viral loads than HAdV-B and HAdV-C infections. Although this may reflect lower replication in the respiratory tract, the small number of HAdV-F cases (n = 4) and the cross-sectional nature of the analysis preclude firm conclusions. Notably, this observation coincides with the circulation of HAdV-F41 during early 2022, the same period as the outbreak of unexplained paediatric hepatitis, where HAdV-F41 and AAV2 were suspected contributors (Marsh et al. 2022, Morfopoulou et al. 2023). However, in the absence of paired stool samples, it was not possible to distinguish between true respiratory infection, concomitant gastrointestinal infection, or prolonged viral shedding. This limitation restricts the interpretation of HAdV-F41 detection in respiratory specimens. Future studies including paired respiratory and stool samples would help better characterize adenoviral tropism and improve interpretation of molecular findings in relation to clinical presentation.

Intrahost diversity findings should be considered exploratory. High read duplication is expected with probe-capture sequencing of viral genomes; however, as duplicates were not removed, support for low-frequency iSNVs may have been inflated. Together with the limited minor-allele read support and strand imbalance observed for some variants, this warrants cautious interpretation of iSNVs near the 5% threshold. Exploratory intrahost diversity analyses revealed substantial within-host genetic variability across respiratory HAdV infections, with higher iSNV counts observed in HAdV-C samples compared with HAdV-B. These findings are consistent with previous reports demonstrating detectable intrahost adenoviral diversity despite the relatively low mutation rate of DNA viruses. Recent studies further suggested that intrahost evolution may contribute to adenoviral adaptation and diversification, particularly during persistent infections [34]. Although our study lacked longitudinal sampling, the biological basis of the higher iSNV counts observed in HAdV-C cannot be established from the present data. Although co-infections involving distinct HAdV strains may occur, we found no evidence of a widespread mixed-population signature. Patterns observed in a small proportion of samples could reflect either low-level mixed infection or technical variation.

The non-random distribution of iSNVs observed in our study is consistent with previous evidence of localized intrahost variation in HAdV. In particular, the recurrent signal identified near the VA RNA locus in HAdV-B overlaps a non-coding hotspot previously reported in HAdV-B3 (Huo et al. 2022) whereas our other major peaks involved different coding and regulatory regions. This partial overlap suggests that some genomic regions may recurrently accumulate intrahost variation, although the distribution of hotspots may vary across viral lineages and epidemiological contexts. Several recurrent signals also occurred in regions known to contribute to adenovirus genomic diversity and recombination, including E3 and capsid-associated genes (Rivailler et al. 2019, Jin et al. 2025). However, the biological significance of these patterns remains uncertain. Higher sequencing depth at recurrent positions does not exclude depth-dependent detection bias, as greater local depth may increase the probability of detecting low-frequency variants. Given the frequent low minor-allele support and strand imbalance, retention of duplicate reads, and potential depth- and mapping-related biases, these findings should be considered descriptive and hypothesis-generating rather than as firm evidence of biologically selected hotspots or diversifying selection, and require confirmation with approaches optimized for low-frequency variant detection.

The main limitation of this study is the lack of detailed clinical data, which limits assessment of the relationship between HAdV type, viral load, and disease severity. Nevertheless, our results underscore the importance of integrating clinical, virological, and genomic data in future research. Clinical outcome-linked viral genomics could improve our understanding of HAdV pathogenesis and support the development of risk-based diagnostics or treatments.

Capture-based whole-genome sequencing may introduce species-dependent biases in sequencing efficiency. A previous study using similar HAdV-specific enrichment probes reported lower genome recovery for HAdV-C viruses despite comparable viral loads and sequencing depth (Houldcroft et al. 2018). Such biases may partly explain why some samples failed confirmation or did not achieve sufficient genome coverage for downstream analyses. However, retained genomes showed high overall coverage, supporting reliable whole-genome phylogenetic analysis and genotype assignment.

Successive filtering steps may have introduced selection bias. In particular, downstream analyses were restricted to mono-infected samples, and the final WGS dataset was enriched for samples with higher HAdV viral loads. Formal comparisons across the main sample selection steps identified limited evidence of selection bias related to age or healthcare center/multiplex assay, except that co-infected patients were younger than mono-infected patients. In contrast, lower viral load was strongly associated with WGS failure, indicating potential selection toward higher viral loads in the sequenced dataset. In addition, exclusion of co-infected samples may potentially have influenced the observed genotype distribution, Previous studies also reported that HAdV-C and, to a lesser extent, HAdV-B species are more frequently associated with respiratory viral co-detection than other species (Mandelia et al. 2021, Probst et al. 2025).

This study enriches our understanding of the epidemiology of HAdVs in France, consistent with global trends while highlighting specific local epidemiological features that necessitate ongoing surveillance and further studies. Our findings confirm high prevalence, age-related virological patterns, and a wide range of circulating genotypes, including recombinant and potentially more virulent variants such as HAdV-B66 and B7d. These observations highlight the dynamic evolution of HAdVs and the importance of continued molecular surveillance, as genotype shifts driven by recombination, mutation, or ecological factors may have significant public health implications. Strengthening genomic monitoring networks will be essential for timely detection of emerging strains and for informing prevention and control strategies.

## Supplementary Material

Supplementary_materials_veag064

## Data Availability

All consensus sequences generated in this study have been deposited in GenBank under accession numbers PX575684–PX575805.
