# Supplementary material for: Molecular epidemiology of paediatric respiratory adenovirus infections in Île-de-France: findings from whole-genome sequencing
Source: Virus Evol. 2026 Sep 26;12(1):veag064. doi: 10.1093/ve/veag064 (PMC13669953; doi:10.1093/ve/veag064)
Supplement: Supplementary file 1 [file supplementary_materials_veag064.zip › Supplementary_materials_veag064.docx]

**Supplementary Table 1. Epidemiological and virological characteristics across the main sample selection steps. Data are presented as median (IQR) or n (%). FilmArray: BioFire® FilmArray RP2 panel; Allplex: Allplex™ Respiratory Panel.**

| **Characteristic** | **Co-infection** | **Mono-infection** | **Mono-infection not quantified** | **HAdV DNA undetectable** | **HAdV DNA <500 copies/mL** | **WGS success** | **WGS failure** |
| --- | --- | --- | --- | --- | --- | --- | --- |
|  | **N = 621** | **N = 350** | **N = 96** | **N = 53** | **N = 57** | **N = 122** | **N = 22** |
| **Age, years median (IQR)** | **1.0 (0.0–2.0)** | **2.0 (1.0–4.0)** | **1.0 (0.0–3.0)** | **4.0 (1.0–11.0)** | **2.0 (0.0–6.0)** | **2.3 (1.1–4.0)** | **2.0 (1.0–6.0)** |
| **Healthcare center / multiplex assay, n (%)** |  |  |  |  |  |  |  |
| **Center 1 (FilmArray)** | **208 (33%)** | **120 (34%)** | **7 (7.3%)** | **29 (55%)** | **31 (54%)** | **46 (38%)** | **7 (33%)** |
| **Center 2 (FilmArray)** | **18 (2.9%)** | **13 (3.7%)** | **1 (1.0%)** | **3 (5.7%)** | **1 (2.0%)** | **8 (6.6%)** | **1 (4.8%)** |
| **Center 3 (FilmArray)** | **136 (22%)** | **62 (18%)** | **1 (1.0%)** | **18 (34%)** | **17 (30%)** | **18 (15%)** | **8 (38%)** |
| **Center 4 (FilmArray)** | **156 (25%)** | **108 (31%)** | **80 (83%)** | **3 (5.7%)** | **5 (9.0%)** | **15 (12%)** | **1 (4.8%)** |
| **Center 5**  **(Allplex)** | **103 (17%)** | **47 (13%)** | **7 (7.3%)** | **0 (0%)** | **3 (5.0%)** | **35 (29%)** | **4 (19%)** |
| **Log10 viral load (copies/mL) median (IQR)** | **NA** | **NA** | **NA** | **NA** | **NA** | **7.20 (6.05–8.51)** | **3.43 (3.12–4.05)** |

**Supplementary Table 2 (Excel file). Sequencing quality metrics and genome coverage characteristics of HAdV whole-genome sequences retained for downstream phylogenetic analyses.**

**Supplementary Table 3. Variant-level quality control metrics for retained intrahost single-nucleotide variants (iSNVs), overall and by HAdV species.**

| **Metric** | **HAdV-B** | **HAdV-C** | **HAdV-F** | **Overall** |
| --- | --- | --- | --- | --- |
| **iSNVs (n)** | 10,649 | 8,773 | 151 | 19,573 |
| **Samples (n)** | 75 | 38 | 4 | 117 |
| **Total depth at site, median [IQR]** | 171 [132-242] | 159 [122-237] | 114 [104-130] | 165 [127-238] |
| **Minor-allele reads, median [IQR]** | 13 [9-22] | 12 [9-19] | 8 [7-9] | 12 [9-21] |
| **iSNVs with >=10 minor reads, %** | 73.4 | 69.3 | 17.2 | 71.1 |
| **MAF, median [IQR]** | 0.068 [0.057-0.093] | 0.068 [0.057-0.089] | 0.064 [0.058-0.078] | 0.068 [0.057-0.091] |
| **iSNVs at MAF 5-10%, %** | 78.3 | 80.8 | 92.7 | 79.5 |
| **Both-strand support, %** | 34.6 | 21.8 | 11.9 | 28.7 |

HAdV, human adenovirus; IQR, interquartile range; iSNV, intrahost single-nucleotide variant; MAF, minor allele frequency

**Supplementary Table 4. Distribution of recurrent intrahost single-nucleotide variant (iSNV) positions across genomic regions.**

*Recurrent positions were defined as genomic positions carrying an iSNV in ≥3 independent samples mapped to the same genotype reference. Overall, 347 recurrent positions were identified, of which 341 were annotated. Maximum recurrence indicates the highest number of samples sharing an iSNV at the same position within each region. Depth represents the median total sequencing depth at recurrent positions.* “Other coding region” includes minor HAdV coding sequences not listed separately.

| **Region** | **Category** | **Recurrent positions (n)** | **Genotypes** | **Max recurrence (samples)** | **Median depth (x)** |
| --- | --- | --- | --- | --- | --- |
| E3 | Early gene | 49 | B3, B7, C1 | 62 | 206 |
| E1A/E1B | Early gene | 33 | B3, C1, C2, C5, C89 | 17 | 188 |
| L4 100K | Late gene | 32 | B3, C1, C2 | 41 | 198 |
| DBP/E2A | Early gene | 29 | B3, B7, C1, C2 | 15 | 241 |
| Other coding region | Coding (other) | 21 | B3, C1, C2, C5, C89 | 7 | 289 |
| VA RNA | Non-coding / regulatory | 20 | B3 | 43 | 250 |
| hexon | Capsid/structural | 19 | B3, C1 | 4 | 169 |
| L1 | Late gene | 17 | B3 | 14 | 148 |
| E4 | Early gene | 16 | B3, C1, C5, C89 | 7 | 224 |
| fiber | Capsid/structural | 12 | B3, C1 | 9 | 229 |
| penton base | Capsid/structural | 12 | B3, B7, C1, C89 | 15 | 223 |
| E2B | Early gene | 5 | B3 | 3 | 263 |
| L2 | Late gene | 1 | B3 | 54 | 1,342 |
| ITR (terminal repeat) | Terminal repeat (mapping caveat) | 75 | B3, C1, C2, C5, C89 | 50 | 178 |
| Unannotated | Unannotated | 6 | B3, C2, C89 | 21 | 168 |

DBP, DNA-binding protein; HAdV, human adenovirus; iSNV, intrahost single-nucleotide variant; ITR, inverted terminal repeat; VA RNA, virus-associated RNA.

**Supplementary Table 5 (Excel file). Variant-level quality-control metrics for retained intrahost single-nucleotide variants (iSNVs).** *One row is provided per retained iSNV, including sample, HAdV species and genotype, reference sequence, genomic position and gene, reference and alternate alleles, total depth, minor-allele read count and frequency, forward- and reverse-strand alternate-read counts, minimum strand support, and strand balance. Strand balance was calculated as min(forward, reverse)/total alternate-allele reads. HAdV, human adenovirus; iSNV, intrahost single-nucleotide variant.*

**Supplementary Figure 1. Overview of the analytical workflow used in the study**

**Supplementary Figure 2. Distribution of human adenovirus (HAdV) DNA viral load (log_10_ copies/mL) by age group.** Boxplots showing the distribution of quantifiable HAdV loads (log_10_ copies/mL) among patients stratified into three age groups: <2 years, 2–5 years, and >5 years. Individual data points are overlaid with jitter. The horizontal bar within each box indicates the median viral load. Values at or above 9 log_10_ copi es/mL correspond to the upper limit of quantification of the assay, viral loads above this threshold were capped at 9 log_10_ copies/mL. No statistically significant differences in HAdV load were observed between age groups (p > 0.05, ns).

**Supplementary Figure 3. Distribution of human adenovirus (HAdV) DNA viral load using uncapped instrument values. (A) Distribution of quantifiable HAdV loads (log10 copies/mL) among patients stratified into three age groups: <2 years, 2–5 years, and >5 years. (B) Distribution of quantifiable HAdV loads (log10 copies/mL) stratified by HAdV species.** Boxplots show the distribution of viral loads, with individual data points overlaid with jitter. The horizontal line within each box indicates the median viral load. Instrument values above the upper limit of quantification of the assay (9 log10 copies/mL) were retained without capping (range, 9.01–10.96 log10 copies/mL). Statistical comparisons between groups are indicated by asterisks, where p<0.05 is denoted by *, p<0.01 by **, p<0.001 by ***, and p<0.0001 by ****; ns indicates no statistically significant difference.

**Supplementary Figure 4. Genome-wide sequencing depth by HAdV species.** The black line represents the median sequencing depth at each genomic position, and the shaded area represents the interquartile range across samples.

**Supplementary Figure 5. Intrahost single nucleotide variant (iSNV) diversity analyses according to HAdV species.** (A) Genome-wide distribution of iSNVs across the adenoviral genome according to HAdV species. Each point represents one iSNV position, with the y-axis indicating minor allele frequency. (B) Nucleotide diversity (π) according to HAdV species. Pairwise comparisons were performed using Wilcoxon rank-sum tests with Benjamini–Hochberg correction for multiple testing. (C) Number of iSNVs detected per sample according to HAdV species. Pairwise comparisons were performed using Wilcoxon rank-sum tests with Benjamini–Hochberg correction for multiple testing.
