# Supplementary material for: Molecular epidemiology of paediatric respiratory adenovirus infections in Île-de-France: findings from whole-genome sequencing
Source: Virus Evol. 2026 Sep 26;12(1):veag064. doi: 10.1093/ve/veag064 (PMC13669953; doi:10.1093/ve/veag064)
Supplement: Supplementary file 1 [file supplementary_materials_veag064.zip › SuppFigure5.pdf]

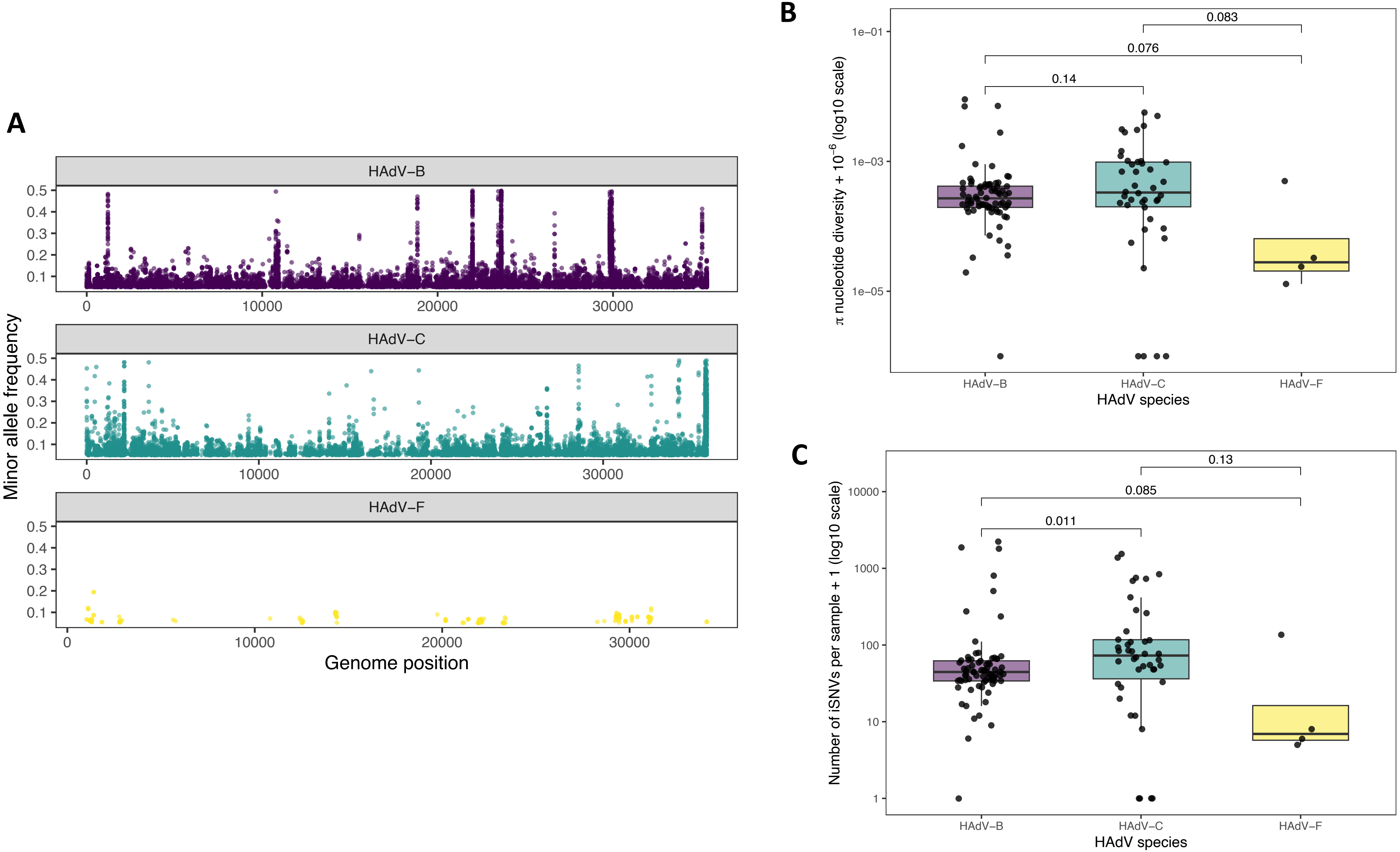
A
HAdV–B
HAdV–C
HAdV–F
0.5
0.4
0.3
0.2
0.1
0
10000
20000
30000
Minor allele frequency
Genome position
B
0.083
0.076
0.14
1e−01
1e−03
1e−05
π nucleotide diversity + 10^−6 (log10 scale)
HAdV–B
HAdV–C
HAdV–F
HAdV species
C
0.13
0.085
0.011
10000
1000
100
10
1
Number of iSNVs per sample + 1 (log10 scale)
HAdV–B
HAdV–C
HAdV–F
HAdV species
