# Supplementary material for: Molecular epidemiology of paediatric respiratory adenovirus infections in Île-de-France: findings from whole-genome sequencing
Source: Virus Evol. 2026 Sep 26;12(1):veag064. doi: 10.1093/ve/veag064 (PMC13669953; doi:10.1093/ve/veag064)
Supplement: Supplementary file 1 [file supplementary_materials_veag064.zip › SuppFigure4.pdf]

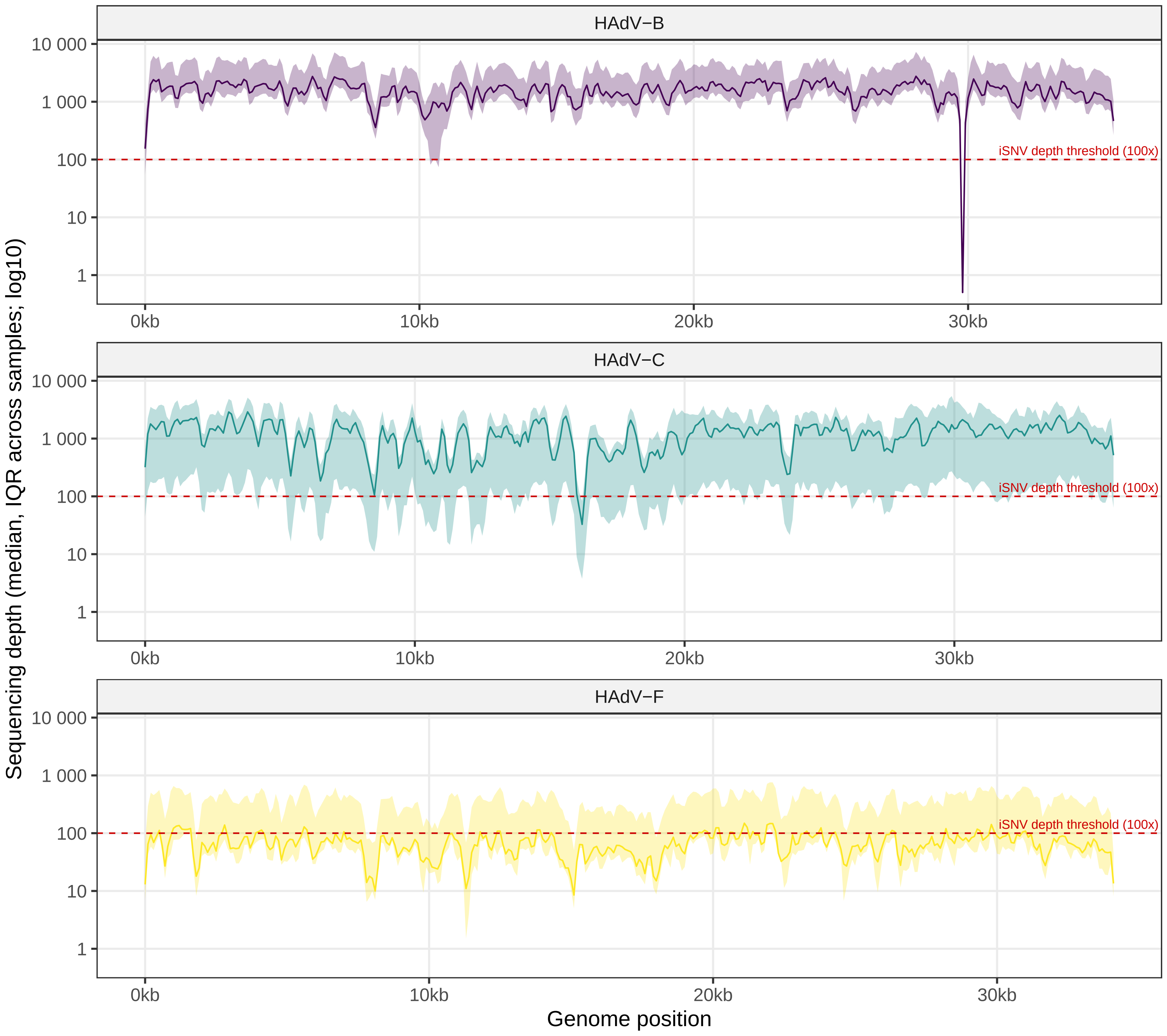

HAdV−B
HAdV−C
HAdV−F
10 000
1 000
100
10
1
iSNV depth threshold (100x)
0kb
10kb
20kb
30kb
Sequencing depth (median, IQR across samples; log10)
Genome position
