# Supplementary material for: Molecular epidemiology of paediatric respiratory adenovirus infections in Île-de-France: findings from whole-genome sequencing
Source: Virus Evol. 2026 Sep 26;12(1):veag064. doi: 10.1093/ve/veag064 (PMC13669953; doi:10.1093/ve/veag064)
Supplement: Supplementary file 1 [file supplementary_materials_veag064.zip › SuppFigure3.pdf]

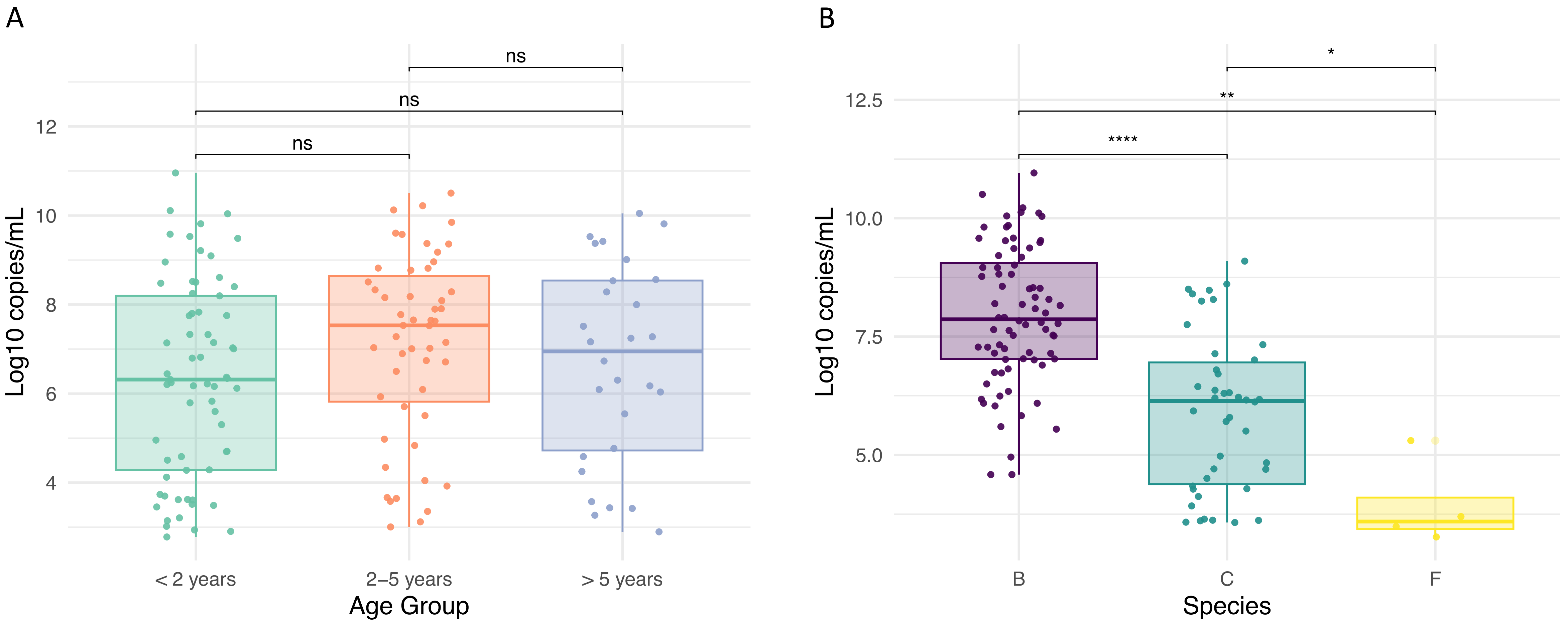
A
ns
ns
ns
Log10 copies/mL
12
10
8
6
4
< 2 years
2–5 years
> 5 years
Age Group
B
*
**
****
Log10 copies/mL
12.5
10.0
7.5
5.0
B
C
F
Species
