# Supplementary material for: Molecular epidemiology of paediatric respiratory adenovirus infections in Île-de-France: findings from whole-genome sequencing
Source: Virus Evol. 2026 Sep 26;12(1):veag064. doi: 10.1093/ve/veag064 (PMC13669953; doi:10.1093/ve/veag064)
Supplement: Supplementary file 1 [file supplementary_materials_veag064.zip › suppFigure2.pdf]

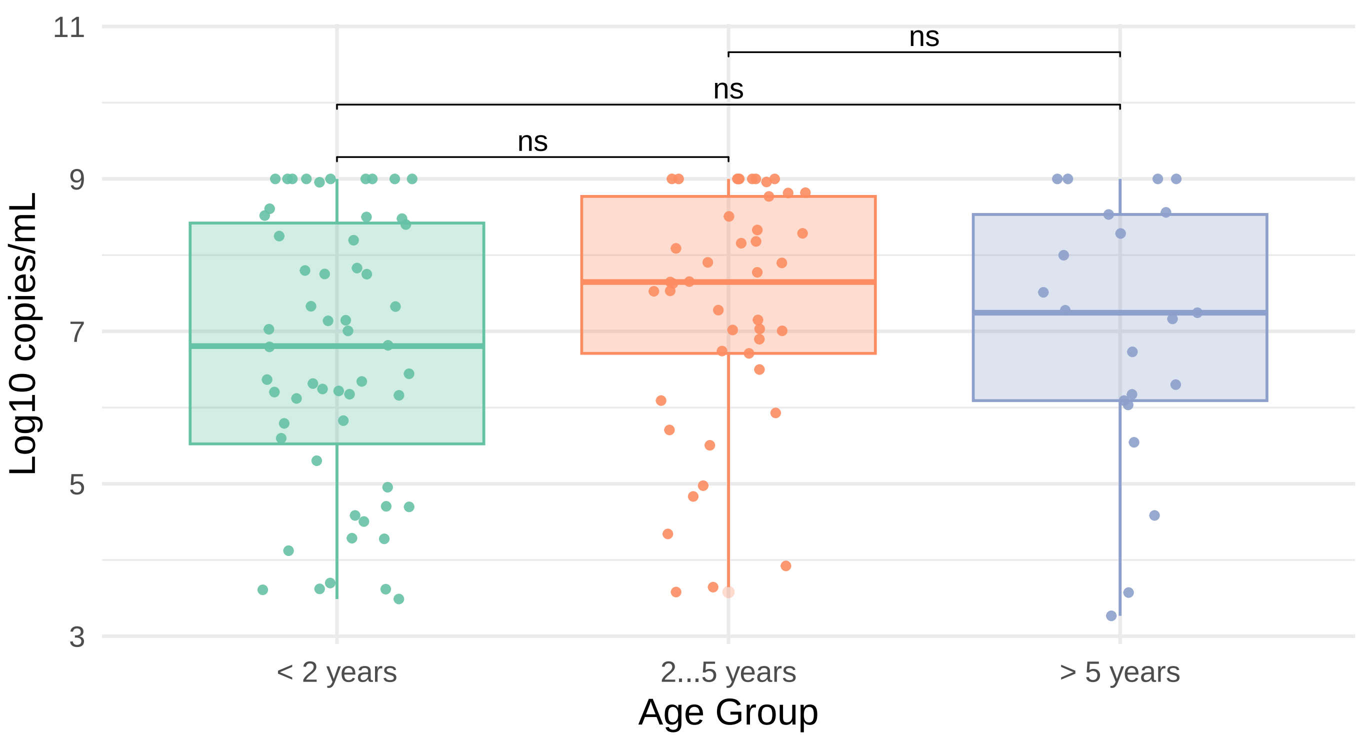

ns
ns
ns
11
9
7
5
3
Log10 copies/mL
< 2 years
2...5 years
> 5 years
Age Group
