# Supplementary material for: Molecular epidemiology of paediatric respiratory adenovirus infections in Île-de-France: findings from whole-genome sequencing
Source: Virus Evol. 2026 Sep 26;12(1):veag064. doi: 10.1093/ve/veag064 (PMC13669953; doi:10.1093/ve/veag064)
Supplement: Supplementary file 1 [file supplementary_materials_veag064.zip › SuppFigure1.pdf]

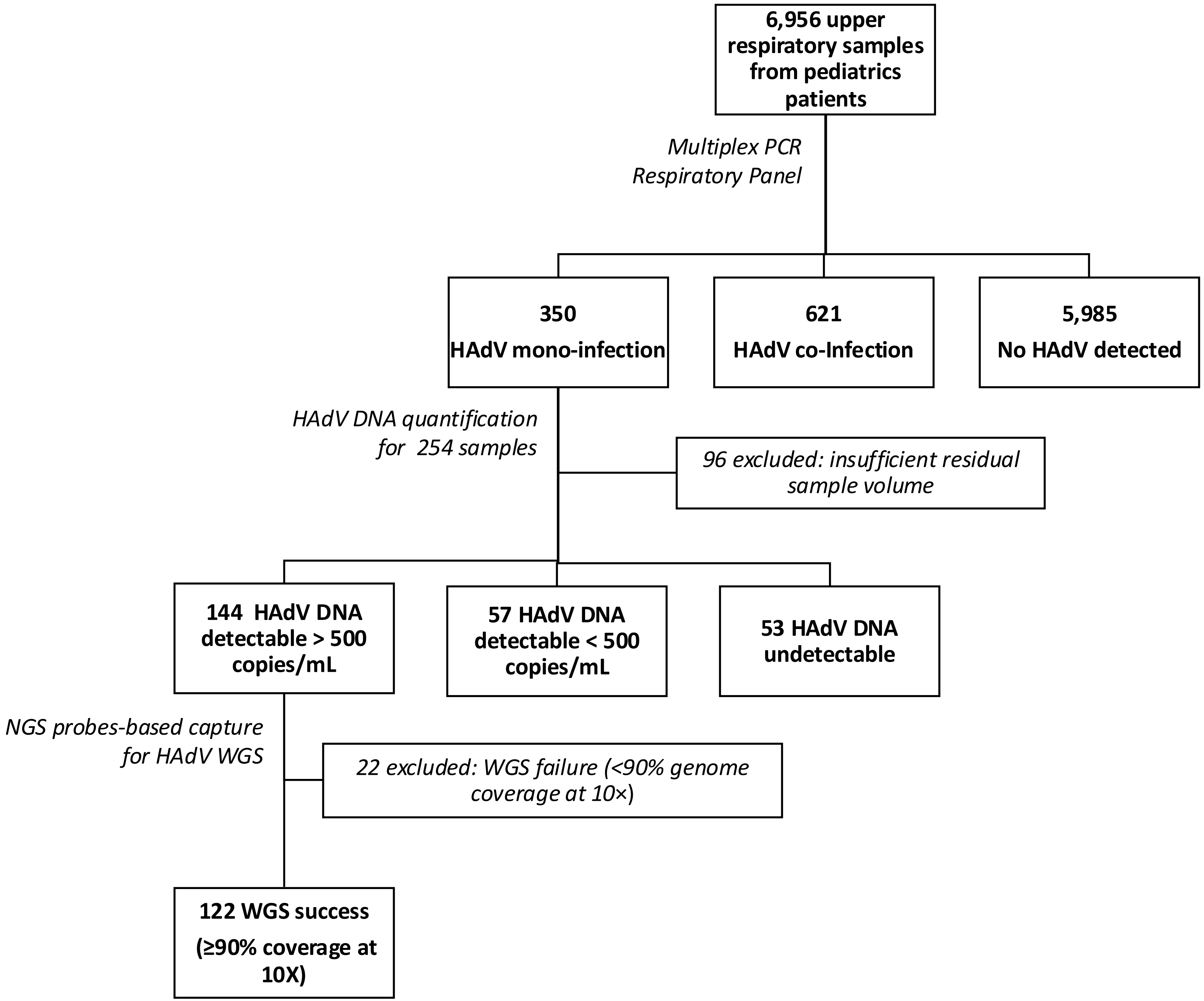
6,956 upper respiratory samples from pediatrics patients
Multiplex PCR Respiratory Panel
350 HAdV mono-infection
621 HAdV co-Infection
5,985 No HAdV detected
HAdV DNA quantification for 254 samples
96 excluded: insufficient residual sample volume
144 HAdV DNA detectable > 500 copies/mL
57 HAdV DNA detectable < 500 copies/mL
53 HAdV DNA undetectable
NGS probes-based capture for HAdV WGS
22 excluded: WGS failure (<90% genome coverage at 10×)
122 WGS success (≥90% coverage at 10X)
